# Disjunctive shared information between ontology concepts: application to Gene Ontology

**DOI:** 10.1186/2041-1480-2-5

**Published:** 2011-08-31

**Authors:** Francisco M Couto, Mário J Silva

**Affiliations:** 1 Departamento de Informática, Faculdade de Ciências da Universidade de Lisboa, Lisboa, 1749-016, Portugal

## Abstract

**Background:**

The large-scale effort in developing, maintaining and making biomedical ontologies available motivates the application of similarity measures to compare ontology concepts or, by extension, the entities described therein. A common approach, known as semantic similarity, compares ontology concepts through the information content they share in the ontology. However, different disjunctive ancestors in the ontology are frequently neglected, or not properly explored, by semantic similarity measures.

**Results:**

This paper proposes a novel method, dubbed DiShIn, that effectively exploits the multiple inheritance relationships present in many biomedical ontologies. DiShIn calculates the shared information content of two ontology concepts, based on the information content of the disjunctive common ancestors of the concepts being compared. DiShIn identifies these disjunctive ancestors through the number of distinct paths from the concepts to their common ancestors.

**Conclusions:**

DiShIn was applied to Gene Ontology and its performance was evaluated against state-of-the-art measures using CESSM, a publicly available evaluation platform of protein similarity measures. By modifying the way traditional semantic similarity measures calculate the shared information content, DiShIn was able to obtain a statistically significant higher correlation between semantic and sequence similarity. Moreover, the incorporation of DiShIn in existing applications that exploit multiple inheritance would reduce their execution time.

## Background

Comparison techniques have always been essential tools for managing knowledge. For example, the study and analysis of a given protein often starts by comparing it with related proteins, and that characterization can be helpful to better understand it. However, the number of possible proteins that can be compared is huge and does not stop growing, due to contemporary high-throughput technologies. Thus, the quest for efficient advanced computational sequence comparison techniques to search for similar proteins is omnipresent in many fields of proteomics.

The most straightforward comparison methods are sequence-based. They only require information on their internal structure (the sequence itself), but limit the analysis to proteins sharing a similar structure, independently of their biological role. This ignores ontological knowledge about the properties and relationships among proteins. For example, when looking for proteins with an oxidoreductase activity, we may be not only interested in proteins annotated with this activity, but also other similar activities, such as monooxygenase activity, independently on how structurally similar the proteins are. Thus, in opposition or as a complement to structural similarity, we should also attempt to compare proteins based on the relationships between them [1].

This has motivated the development of ontology-based similarity measures in the past [2], defining similarity between concepts as a combination of the measures of their common and distinctive relationships, inspired on Tversky's contrast model [3]. Ontology-based similarity has become a prominent approach to compare biomedical entities based on their biomedical activity. Many similarity measures have been applied to biomedical ontologies, and compared against traditional structural similarity measures [4-8]. In the biomedical field, ontology-based similarity measures are normally referred to as *semantic similarity measures*, contrasting with structural similarity measures, and thus this paper also adopts that nomenclature.

Measures based on the information content that two concepts share were the first to identify a correlation between protein sequence similarity and semantic similarity [9]. More recently, the notion of shared information content has been applied to semantically compare diseases, phenotypes and chemical compounds [10-12]. Most ontologies represent relationships between their concepts as Directed Acyclic Graphs (DAG). Thus, the shared information between two concepts is normally proportional to the information content of the Most Informative Common Ancestor (MICA) in the DAG, and the Information Content (IC) of a concept is inversely proportional to its frequency in a given corpus. The frequency of a concept is also propagated to its ancestors, making the IC of a concept related to its depth in the DAG. When entity mappings are available, frequency is normally defined as the number of entities mapped to each concept, normally referred to as annotations.

**Figure 1:**
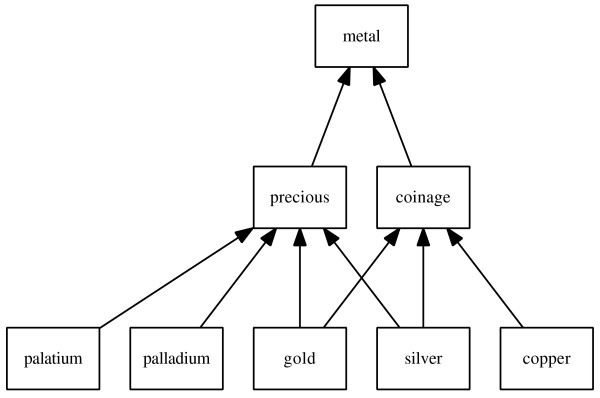
**Classification of metals**. This DAG represents an example of a classification of metals with multiple inheritance, since *gold *and *silver *are considered both precious and coinage metals.

For example, considering the DAG represented in Figure 1 and assuming a non-zero frequency for each concept, the IC of *copper *will always be higher than the IC of *coinage*, which in turn will be higher than the IC of *metal*. Therefore, a semantic similarity between *copper *and *gold *is proportional to the IC of *coinage*, their MICA, and the similarity between *copper *and *palatium *is proportional to the IC of *metal*, their MICA. As expected, this means that, independently of the frequency calculation, the similarity between *copper *and *gold *will be higher than the similarity between *copper *and *palatium*.

Using only the MICA to define similarity equates to considering the DAG as a tree, i.e. neglecting the multiple inheritance nature of the DAG. This problem was identified by Resnik, who decided to use only one of the possibilities for each concept [13]. The decision is consistent with previous treatments of disjunctive concepts [14], where they define the distance between two disjunctive sets of concepts as the minimum path length from any element of the first set to any element of the second. Despite the value of this approach in natural language processing applications, in other domains, such as the Life Sciences, similarity measures are expected to account for the multi-faceted nature of their concepts and entities. The exploitation of multiple inheritance was previously addressed by GraSM, where the shared information content between two concepts is re-defined as the average of all their disjunctive ancestors [15]. GraSM assumes that two common ancestors are disjunctive if there are independent paths from both ancestors to each concept. The implementation of GraSM is rather complex, and it lowers the similarity of concepts that share parallel interpretations instead of raising it, as this represents a stronger relation between concepts sharing more independent information. Taking the example in Figure 1, GraSM considers *platinum *and *palladium *more similar than *platinum *and *gold*, since *gold *can have a different interpretation (coinage). However, we could also expect that *silver *and *gold *to be more similar than *platinum *and *gold *or *platinum *and *palladium*, since *silver *and *gold *share two parallel interpretations, *precious *and *coinage*. GraSM considers the opposite, since *silver *and *gold *have two interpretations, it reduces their similarity, which is counterintuitive.

To overcome the problems described above, this paper proposes a novel method for calculating the shared information content between two concepts, dubbed Disjunctive Shared Information (DiShIn), based on the number of distinct paths between the concepts and their common ancestors. Like GraSM, DiShIn re-defines the shared information content between two concepts as the average of all their disjunctive ancestors. However, DiShIn assumes that an ancestor is disjunctive if the difference between the number of distinct paths from the concepts to it is different from that of any other more informative ancestor. In other words, a disjunctive ancestor is the most informative ancestor representing a given set of parallel interpretations. Like GraSM, DiShIn can be directly integrated into any semantic similarity measure based on the MICA. Taking again the example of Figure 1, DiShIn still considers *platinum *and *palladium *more similar than *platinum *and *gold*. This happens because the number of distinct paths from both *platinum *and *palladium *to *precious *and *metal *is one. Therefore, only *precious *is considered to be a disjunctive ancestor. On the other hand, the number of distinct paths from both *platinum *and *gold *to *precious *is one but from *gold *to *metal *is two. Therefore, both *precious *and *metal *are considered to be disjunctive ancestors. Since the shared information is defined as the average of the disjunctive ancestors and the IC of *metal *is smaller than *precious*, then the similarity between *platinum *and *palladium *is higher than *platinum *and *gold*. However, unlike GraSM, DiShIn does not consider *silver *and *gold *less similar than *platinum *and *gold *or *platinum *and *palladium*. This happens because the number of distinct paths from both *silver *and *gold *to *precious *and *coinage *is one and to *metal *is two. All ancestors have the same number of distinct paths from each concept, thus only *precious *or *coinage *will be considered a disjunctive ancestor, depending of which has the highest IC. This means that the similarity between *silver *and *gold *will be higher than *platinum *and *gold *and at least equal to *platinum *and *palladium*.

We applied DiShIn to one of most popular ontologies in the biomedical domain, the Gene Ontology. The performance of DiShIn was evaluated using CESSM, an existing platform for collaborative and automated evaluation of protein similarity measures [16]. For a pre-defined list of pairs of proteins, CESSM calculates the correlation coefficients between semantic and sequence similarity. Sequence similarity is considered here the golden standard, following the common assumption that entities that are globally similar in structure tend to have similar biological activity [9]. DiShIn was able to obtain statistically significant higher correlation coefficients than GraSM and MICA alone.

Thus, the main contributions of this paper are:

• formalization of a novel method, DiShIn, to calculate shared information content using multiple inheritance (Methods Section);

• application of DiShIn to Gene Ontology (Gene Ontology Application Section);

• evaluation of DiShIn performance against state-of-the-art methods (Results and Discussion Section).

## Methods

This section presents the current approaches to define similarity between ontology concepts as a combination of their common and distinctive relationships in the ontology.

### Semantic similarity

Resnik defined the similarity between two concepts *c*_1 _and *c*_2_, represented as nodes in a DAG, as the amount of information content they share. Given the frequency *freq*(*c*) for each concept *c *in a corpus, the information content of a concept is inversely proportional to the frequency of that concept and its descendants [13]:

$$IC\left(c\right)=-\log\left(\frac{freq\left(c\right)}{maxFreq}\right)$$

where *maxFreq *represents the maximum frequency of all concepts, i.e. the frequency of the root concept when it exists. Then, Resnik defined the amount of information content they share as:

$$Share_{mica}\left(c_{1},c_{2}\right)=max\left\{IC\left(a\right):a\in CA\left(c_{1},c_{2}\right)\right\}$$

where *CA *represents the common ancestors of *c*_1 _and *c*_2_:

$$CA\left(c_{1},c_{2}\right)=Anc\left(c_{1}\right)\cap Anc\left(c_{2}\right)$$

and *Anc*(*c*) represents the set of ancestors of a concept *c*. Resnik's similarity measure only uses the IC of a single common ancestor, the most informative one, the MICA.

$$Sim_{resnik}\left(c_{1},c_{2}\right)=Share_{mica}\left(c_{1},c_{2}\right)$$

Jiang and Conrath defined distance between concepts as the difference between the ICs of both concepts and the IC of their MICA [17]:

$$\begin{array}{c} Dist_{jc}\left(c_{1},c_{2}\right)=\\ \;\;\;IC\left(c_{1}\right)+IC\left(c_{2}\right)-2\times Share_{mica}\left(c_{1},c_{2}\right) \end{array}$$

Lin defined similarity as the IC of their MICA over the IC of both concepts [18]:

$$Sim_{lin}\left(c_{1},c_{2}\right)=\frac{2\times Share_{mica}\left(c_{1},c_{2}\right)}{IC\left(c_{1}\right)+IC\left(c_{2}\right)}$$

All of these measures defined similarity or distance based on the same Resnik definition of shared information that uses a single common ancestor. To deal with multiple inheritance, Couto et al. proposed GraSM, a new definition of shared information [15]. GraSM defines it as the average of the information content of the disjunctive common ancestors of both concepts:

$$\begin{array}{c} Share_{grasm}\left(c_{1},\;c_{2}\right)=\\ \;\;\;\bar{\left\{IC\left(a\right):\;a\in DCA_{grasm}\left(c_{1},\;c_{2}\right)\right\}} \end{array}$$

where *DCA_grasm _*represents the disjunctive common ancestors of both concepts:

$$\begin{array}{l} DCA_{grasm}(c_{1},\;c_{2})=\{a_{1}|\\ a_{1}\in CA(c_{1},\;c_{2})\wedge \forall a_{2}:\\ \left(a_{2}\in CA(c_{1},\;c_{2})\wedge IC(a_{1})\leq IC(a_{2})\wedge a_{1}\neq a_{2}\right)\\ \Rightarrow ((a_{1},\;a_{2})\in DA_{grasm}(c_{1})\cup DA_{grasm}(c_{2}))\} \end{array}$$

where *DA_grasm _*represents the disjunctive ancestors of a concept:

DAgrasm(c)={(a1, a2)|(∃p:p∈Paths(a1, c)∧a2∈p)∧(∃p:p∈Paths(a2, c)∧a1∈p)}

where *Paths*(*a, c*) gives the set of distinct paths from *c *to *a *in the DAG.

For GraSM, a disjunctive common ancestor is an ancestor for which there is a path from one of the concepts to that ancestor, distinct of any other path from that same concept to the other disjunctive common ancestors. This recursive definition makes the computational complexity of its implementation non-linear, which strongly limits its potential for integration in large-scale studies. Moreover, GraSM decreases the shared information even when two disjunctive common ancestors represent two parallel interpretations shared by both concepts, such as the case of *silver *and *gold *of Figure 1, where GraSM defines the disjunctive common ancestors as:

$$\begin{array}{c} DCA_{grasm}\;\left(platinum,\;palladium\right)\;=\;\left\{precious\right\}\\ DCA_{grasm}\;\left(silver,\;gold\right)\;=\;\left\{precious,\;coinage\right\} \end{array}$$

since there are distinct paths both from *silver *and *gold *to *precious *and *coinage*. Then, GraSM defines their shared information as:

$$\begin{array}{c} Share_{grasm}\;\left(platinum,\;gold\right)\;=IC\left(precious\right)\\ \;\;Share_{grasm}\;\left(silver,\;gold\right)\;=\\ \;\;\;\;\;\;\frac{IC\left(precious\right)+IC\left(coinage\right)}{2} \end{array}$$

Thus, in the case where *IC*(*precious*) *> IC*(*coinage*) we will have

$$\begin{array}{c} Share_{grasm}\;\left(silver,\;gold\right)\;<\\ \;\;\;Share_{grasm}\;\left(platinum,\;palladium\right) \end{array}$$

In the case where *IC*(*precious*) *< IC*(*coinage*) we will have the opposite, but *Share_grasm_*(*silver, gold*) will still be penalized against any other pair of concepts that only share *coinage*.

### Proposed approach

To overcome the limitations of GraSM, this paper proposes DiShIn, a new definition of shared information that re-defines the disjunctive common ancestors as:

$$\begin{array}{l} DCA_{DiShIn}(c_{1},\;c_{2})=\{a:\\ a\in CA(c_{1},\;c_{2})\wedge \\ \forall_{a_{x}\in CA(c_{1},c_{2}}{}_{)}PD(c_{1},\;c_{2},\;a)=PD(c_{1},\;c_{2},\;a_{x})\\ \Rightarrow IC(a)>IC(a_{x})\} \end{array}$$

where *CA *represents the common ancestors and *PD *the difference between the number of paths from the two concepts to their ancestor:

$$PD\left(c_{1},\;c_{2},\;a\right)=|Paths\left(c_{1},\;a\right)-Paths\left(c_{2},\;a\right)|$$

where *Paths *gives the number of distinct paths from *c *to *a *in the DAG.

Therefore, the shared information between two concepts can be defined as:

$$\begin{array}{c} Share_{dishin}\left(c_{1},\;c_{2}\right)=\\ \;\;\;\bar{\left\{IC\left(a\right):\;a\in DCA_{dishin}\left(c_{l},\;c_{2}\right)\right\}} \end{array}$$

As in GraSM, DiShIn can be integrated in any other semantic similarity measure based on shared information content:

$$\begin{array}{c} \;\;Si{m_{resnik:}}_{dishin}\;\left(c_{1},\;c_{2}\right)=Share_{dishin}\left(c_{1},\;c_{2}\right)\\ Dis{t_{jc:}}_{dishin}\;\left(c_{1},\;c_{2}\right)=\\ \;\;\;IC\left(c_{1}\right)+IC\left(c_{2}\right)-2\times Share_{DiShIn}\left(c_{1},\;c_{2}\right)\\ \;\;Sim_{lin:dishin\;}\left(c_{1},\;c_{2}\right)=\frac{2\times Share_{dishin}\left(c_{1},c_{2}\right)}{IC\left(c_{1}\right)+IC\left(c_{2}\right)} \end{array}$$

#### Example

To illustrate how DiShIn handles parallel interpretations differently from GraSM, this section presents the application of DiShIn to the case of multiple inheritance of Figure 1.

DiShIn starts by calculating the path difference for all the common ancestors of the pairs (*platinum, palladium*), (*platinum, gold*) and (*silver, gold*):

$$\begin{array}{cc} PD\;\left(platinum,\;palladium,\;precious\right) & =\;|1-1|\;=0\\ PD\;\left(platinum,\;palladium,\;metal\right) & =\;|1-1|\;=0\\ PD\;\left(platinum,\;gold,\;precious\right) & =\;|1-1|\;=0\\ PD\;\left(platinum,\;gold,\;metal\right) & =\;|1-2|\;=1\\ PD\;\left(silver,\;gold,\;precious\right) & =\;|1-1|\;=0\\ PD\;\left(silver,\;gold,\;coinage\right) & =\;|1-1|\;=0\\ PD\;\left(silver,\;gold,\;metal\right) & =\;|2-2|\;=0 \end{array}$$

This means that there is only a non-zero number of paths from *platinum *and *gold *to *metal *representing the multiple inheritance of *gold *as *coinage *and as *precious*, in opposition to the single inheritance of *platinum*. Note that the difference on the number of paths from *silver *and *gold *to *metal *remains zero, since their multiple inheritance is parallel. Given that *IC*(*precious*) *> IC*(*metal*) and *IC*(*coinage*) *> IC*(*metal*), DiShIn defines the common disjunctive ancestors of the above pairs of concepts as:

$$\begin{array}{c} \;DCA_{dishin}\;\left(platinum,\;palladium\right)\;=\;\left\{precious\right\}\\ \;DCA_{dishin}\;\left(platinum,\;gold\right)\;=\;\left\{precious,\;metal\right\}\\ DCA_{dishin}\;\left(silver,\;gold\right)\;=\\ \;\;\;\left\{\begin{array}{cc} \left\{coinage\right\} & if\;IC\;\left(precious\right)\;<IC\;\left(coinage\right)\\ \left\{precious\right\} & otherwise \end{array}\right) \end{array}$$

Only (*platinum, gold*) has two common disjunctive ancestors given their different number of paths to *metal*. The shared information content is then calculated by averaging the IC of their common disjunctive ancestors:

$$\begin{array}{c} Share_{dishin}\;\left(platinum,\;palladium\right)\;=IC\;\left(precious\right)\\ \;\;\;\;\;\;Share_{dishin}\;\left(platinum,\;gold\right)\;=\\ \;\;\;\;\;\;\;\;\;\frac{IC\left(precious\right)+IC\left(metal\right)}{2}\\ \;\;\;Share_{dishin}\;\left(silver,\;gold\right)\;=\\ \;\;\;\;\;\;max\left\{\;IC\;\left(precious\right),\;IC\;\left(coinage\right)\right\} \end{array}$$

Unlike in GraSM, we can verify that (*silver, gold*) is not penalized by an average, on the contrary, it gets the maximum IC of their parallel interpretations. This means that we have, as expected:

$$\begin{array}{c} Share_{dishin}\;\left(silver,\;gold\right)\;\geq \\ \;\;\;Share_{dishin}\;\left(platinum,\;palladium\right)\;>\\ \;\;\;Share_{dishin}\;\left(platinum,\;gold\right) \end{array}$$

This shows that, unlike GraSM, DiShIn does not penalize pairs of concepts with parallel interpretations, and, like GraSM, it penalizes pairs of concepts with distinct paths for the same interpretation.

#### Computation

Before using DiShIn, we need to estimate the IC for each concept, and calculate the number of distinct paths from one concept to another, *Paths*(*c*_1_, *c*_2_). These preliminary calculations depend on the used ontology and on the available annotations. In the worst-case scenario, we need to use an all-pairs shortest paths algorithm to calculate *Paths*(*c*_1_, *c*_2_) and propagate the frequency of concepts to obtain their IC, so we can estimate a computational complexity of $O\left(n^{3}\right)$ for these preliminary calculations, where *n *is the number of ontology concepts [19]. However, the calculations only need to be performed once, and updated as new versions of the ontology become available. Thus, the time spent on these calculations has no impact on the performance of DiShIn.

After calculating the *IC*(*c*) and *Paths*(*c*_1_, *c*_2_), let's assume that we store their information in a relational database as two tables, IC and Paths, respectively. The table IC is composed of two columns, holding the concept identifier and a value representing the information content of the concept. The table Paths is composed of three columns, holding a concept identifier, another concept identifier representing an ancestor of the former concept, and a value representing the number of paths between the two concepts. Thus, with these two tables DiShIn could be implemented as a single SQL query:

**SELECT AVG**(DCA. **value**)

FROM

 (**SELECT MAX**(IC. **value**) **as value**

 **FROM **IC,

   (**SELECT **p1. ancestor **as **ancestor,

         ABS (p1. **value ***- *p2. **value**)

            **as value**

   **FROM **Paths p1, Paths p2

   **WHERE **p1. concept = c1

      **AND **p2. concept = c2

      **AND **p1. ancestor = p2. ancestor

      **AND **p1. **value***>*0 **AND **p2. **value***>*0

   ) **as **PD

 **WHERE **IC. concept = PD. ancestor

 **GROUP BY **PD. **value**

) **as **DCA;

The SQL query contains a subquery that calculates the value of *P D*(*c*_1_, *c*_2_, *a*) according to the values of *P aths*(*c*_1_, *a*) and *P aths*(*c*_2_, *a*) for each *a *∈ *CA*(*c*_1_, *c*_2_). Note that the constraint *a *∈ *CA*(*a*_1_, *a*_2_) is implemented by checking that *Paths*(*c*_1_, *a*) *>*0 and *Paths*(*c*_2_, *a*) *>*0. Next, another subquery groups the results of the previous query by the *PD*(*c*_1_, *c*_2_, *a*) values, and selects the most informative ancestor of each group, which represents the common disjunctive ancestors: *DCA_dishin_*(*c*_1_, *c*_2_). Finally, the query calculates the average of the information content values, i.e. the shared information: *Share_dishin_*(*a*_1_, *a*_2_)

The first subquery returns one row for each common ancestor, so the number of rows returned is limited to *n*. The usage of indexes on table *Paths *enables the computation of this subquery in constant time. Since the other subqueries only perform group and average operations over the rows returned by the first subquery, the computational complexity of the SQL query that implements DiShIn is O(n). Note that the SQL query is universal to any ontology structured as a DAG, only the preliminary calculation of IC and Paths are dependent on the ontology used.

## Gene Ontology application

Semantic similarity measures have been applied to Gene Ontology (GO), a popular biomedical ontology [20], mainly to compare genes or proteins based on the similarity of their activities (modelled as GO concepts).

**Figure 2:**
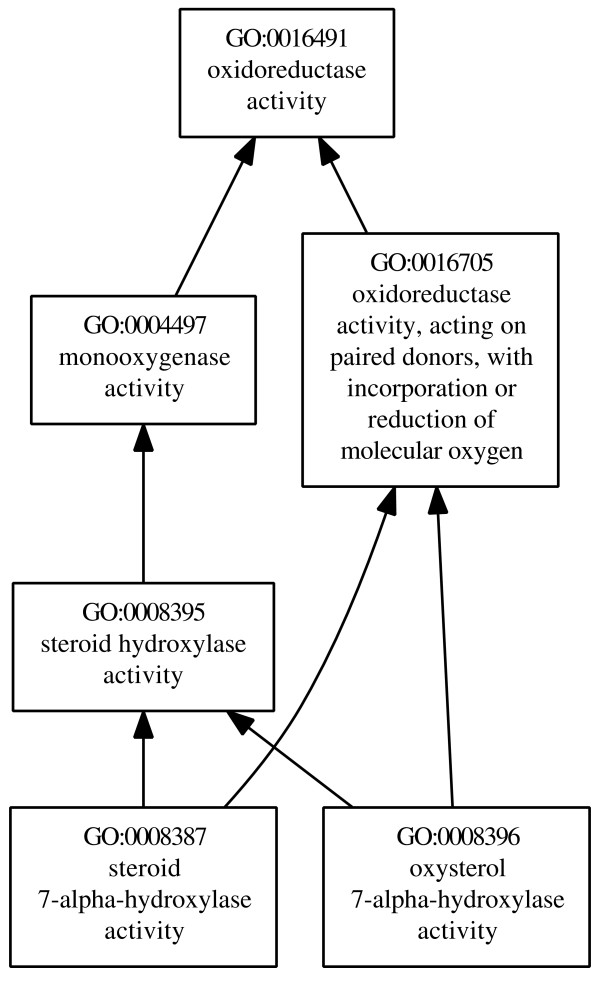
**Example of multiple inheritance in GO**. Subgraph of the *molecular function *subontology of GO containing the common ancestors of the concepts *steroid 7-alpha-hydroxylase activity*, and *oxysterol 7-alpha-hydroxylase activity*.

GO organizes its concepts in three distinct DAGs representing the following subontologies: *molecular function*, *biological process *and *cellular component*. The relations between the concepts have the following types: *is-a*, *part-of *and *regulates*. Semantic similarity measures are normally restricted to *is-a *and/or *part-of *relations, which are required to define the ancestors and descendants of any concept. These relations fit our method requirements. Figure 2 shows an example of the GO hierarchy.

### Preliminary calculations

The IC was estimated using the same approach used by most measures applied to GO [7], where the frequency of a given concept is calculated by counting the number of proteins annotated with it or with any of its descendants in the DAG. Together with the ontology, the GO consortium also provides publicly available releases of these GO annotations.

GO also provides the transitive closure of each DAG, which was used for calculating the number of distinct paths between any pair of concepts. The calculation was performed for all pairs of concepts connected through the transitive closure. Every pair of concepts directly connected in the DAG was considered to have only one distinct path between them. And for every pair of concepts not directly connected, it was identified an intermediate concept directly connected to one of the concepts and whose number of paths to the other concept was already calculated.

### Evaluation platform

A commonly used approach for evaluating semantic similarity measures in biomedical ontologies is based on comparing their correlation with structural similarity. This correlation may not be always accurate, but this approach represents a comprehensive analysis, since structural similarity is present everywhere in Molecular Biology. For example, even functional classifications, like PFAM, rely mostly on structural similarity methods [21]. Therefore, this evaluation assumes that on average the results obtained from a large number of examples should be close to their real value, even if some exceptions exist. A systematic difference between semantic and structural similarity would undermine this assumption, but this is not expected to exist under the assumed correlation between protein function and its structure [22].

Recent studies on GO similarity have used CESSM, a platform that supports the collaborative and automated evaluation of similarity measures based on GO [16]. CESSM provides an unbiased comparison of novel similarity measures against several existing ones by testing them on the same task and data, and then calculating the same performance indicators. The data are composed of a list of protein pairs, a specific release of GO and protein annotations; the task is comparing proteins; and the performance indicators are the correlation coefficients between semantic and sequence similarity.

CESSM provides a list of UniProt protein pairs which have been selected based on their quality of GO annotations, and indicates a specific release of GO and UniProt [23] in which the similarity should be based on. In January of 2011, CESSM was using the August of 2008 release of GO and GO-UniProt datasets and provided a list of 13,430 proteins pairs. For these proteins, we have an average of 5.9 GO annotations per protein in the *Biological Process*, 2.9 in the *Cellular Component*, and 3.7 in the *Molecular Function*. Thus, the DiShIn's pre-processing described above was performed over these datasets, using all protein annotations they contained (manual and electronic).

Semantic similarity measures enable a quantitative comparison between ontology concepts, but not directly between the entities annotated with them, such as proteins. To calculate protein similarity some specialized graph matching measures have been proposed, such as *simGIC *[7], but, by extension, semantic similarity measures can also be adapted to compare the entities mapped to the concepts. This adaptation has to result from combining the similarity of the concepts that the entities are mapped to. Note that an entity, such as a protein, may be mapped to multiple concepts, since proteins are usually involved in multiple biological activities. The most effective adaptation approach is composite (best-match) averages, where each concept of the first protein is paired only with the most similar concept of the second one and vice-versa [24-26]. Thus, for this study, DiShIn adopted this approach to work as a protein similarity measure.

After uploading the similarity values for each measure, CESSM provides the Pearson's linear correlation with sequence similarity [27], a popular approach for comparing proteins and for evaluating GO similarity measures [9]. Therefore, this study used CESSM to obtain the correlation coefficients for the measures: *Sim_resnik_*, *Sim_lin_*, *Dist_jc_*, *Sim*_*resnik*:*dishin *_and *Sim*_*resnik*:*grasm*_, all adapted as protein similarity measures by using the best-match approach.

## Results and discussion

### Pearson's linear correlation

Table 1 presents the values returned by CESSM representing the Pearson's linear correlation between sequence similarity and the similarity obtained by the GO-based measures. Since GO is composed of three distinct subontologies, CESSM calculates the correlation for each one of them separately. Note that all the correlation coefficients were calculated using 13,430 protein similarity values, one for each protein pair in the CESSM dataset.

**Table 1:** Pearson's correlation coefficients

	Resnik	GraSM	p-value	DiShIn	p-value
*Molecular Function*	0.6683	0.6690	0.8923	0.6812	0.0091
*Biological Process*	0.7397	0.7417	0.6133	0.7589	0.00001
*Cellular Component*	0.7113	0.7129	0.7061	0.7268	0.0008

Pearson's linear correlation between semantic and sequence similarity for the 13,430 protein pairs. The p-values represent the probability of obtaining the correlation coefficients for GraSM assuming the correlation coefficients of Resnik, and for DiShIn assuming the correlation coefficients of GraSM.

**Figure 3:**
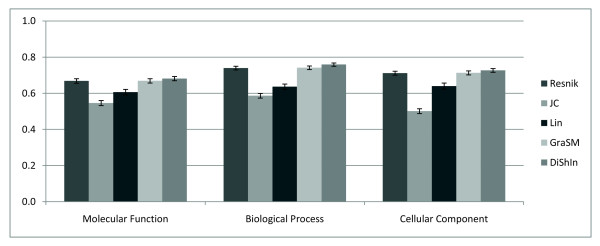
**Pearson's linear correlation**. Pearson's linear correlation between sequence similarity and GO-based measures. In the y-axis we have the correlation values and in the x-axis the GO-based measures in each subontology of GO. The error bars represent the 99% confidence interval.

In Figure 3, for each subontology of GO, *Sim*_*resnik*:*dishin *_provides the highest correlation coefficients and *Sim_lin _*and *Dist_jc _*provide the lowest correlation coefficients. These results show that in this study a more accurate calculation of the shared information content is more relevant than including the IC of the concepts being compared.

Using Fisher's transformation and a one-sample z test, Table 1 presents the p-values for the correlation coefficients of *Sim*_*resnik*:*grasm *_and *Sim*_*resnik*:*dishin*_, considering the null hypothesis as that these coefficients being equal to the coefficients of *Sim_resnik _*and *Sim*_*resnik*:*grasm*_, respectively [[28], eq. 11.22]. Fisher's parametric statistics has been used by many GO applications to measure the significance of obtained results [29], including previous semantic similarity studies [30].

For each subontology of GO, *Sim*_*resnik*:*dishin *_presents a statistically significant increase of the correlation coefficients (p-value *<*0.01), as opposed to the low statistical significance of the increase obtained by *Sim*_*resnik*:*grasm *_(p-value *>*0.6). Using also Fisher's transformation, Table 2 presents the confidence levels for the correlation coefficients of *Sim*_*resnik*:*dishin *_[[28], eq. 11.23]. For example, in the *Biological Process *subontology, at the confidence level of 98%, the lower limit of the confidence interval of the correlation coefficients of *Sim*_*resnik*:*dishin *_is larger than the higher limit of the confidence interval of the correlation coefficients of *Sim_resnik _*and *Sim*_*resnik*:*grasm*_. The different confidence levels of *Sim*_*resnik*:*dishin *_between the three subontologies can be explained by the edge density of each DAG: 1.95 in the *Biological Process*, 1.85 in the *Cellular Component*, and 1.16 in the *Molecular Function*. More edges per node means a higher presence of multiple inheritance, and therefore a higher possibility of the application of DiShIn affecting more similarity calculations.

**Table 2:** Confidence level on Pearson's correlation coefficients

	GraSM/Resnik	DiShIn/Resnik	DiShIn/GraSM
*Molecular Function*	5%	83%	81%
*Biological Process*	20%	99%	98%
*Cellular Component*	15%	94%	90%

The maximum confidence levels that result in non-overlapped confidence intervals for the correlation coefficients of GraSM when compared to Resnik, and for the correlation coefficients of DiShIn when compared to GraSM and Resnik.

*Sim*_*resnik*:*dishin *_was able to improve correlation because it managed to calculate the shared information in a more effective manner than *Sim*_*resnik*:*grasm *_and *Sim_resnik_*. The increase is even more relevant if we take into account that multiple inheritance only affects about 10% of the GO similarity calculations. For example, we only had 5,530 out of 513,850 similarity calculations performed in the *Molecular Function *subontology, with *Sim_resnik _*, *Sim*_*resnik*:*dishin*_. However, the best-match approach averages the GO similarity values obtained by combining the GO concepts annotated with both proteins, where a single GO similarity change may affect the final protein similarity value. Since the proteins in the CESSM dataset are all well annotated, multiple inheritance affected most of similarity values of the 13,430 protein pairs; more specifically this happened in 95% of the proteins pairs in the *Biological Process*, 93% in the *Cellular Component*, and 75% in the *Molecular Function*. Note that these percentages are also coherent with the edge density of each subontology, as described above. For example, in the *Molecular Function *subontology using only the 75% of the proteins pairs that were affected by multiple inheritance drops the correlation coefficients of *Sim_resnik_*, *Sim*_*resnik*:*dishin *_and *Sim*_*resnik*:*dishin *_to 0.4008, 0.4024 and 0.4149, respectively. *Sim*_*resnik*:*dishin *_still presents a significant improvement, but the lower coefficients indicate that proteins with multiple inheritance tend to have a complex biological role that is not so well correlated with sequence similarity. Nonetheless, in 10% of the cases where multiple inheritance exists, *Sim*_*resnik*:*dishin *_managed it in a much more effective way than *Sim*_*resnik*:*grasm *_in order to have achieved the overall improvement of correlation presented above. This also corroborates the hypothesis that multiple inheritance, even if scarce, can have an important overall impact, as previously proposed for GraSM. Hence, when multiple inheritance exists, it should not be neglected, as in the Resnik approach based only on the most informative common ancestor.

### Example

To exemplify how DiShIn differs from GraSM, this section discusses their values when comparing the leaf concepts of Figure 2, *steroid *and *oxysterol 7-alpha-hydroxylase activity*.

According to GraSM, these concepts have two disjunctive common ancestors: *oxidoreductase with oxygen *and *steroid hydroxylase*, whose IC in this study was 0.3846 and 0.6671, respectively. Thus, GraSM returns the average of their IC, $Sim_{resnik:grasm}=\frac{0.6671+0.3846}{2}=0.5259$

On the other hand, for each common ancestor, the number of paths from *steroid *to that ancestor is always equal to the number of paths from *oxysterol *to that same ancestor. For example, the top *oxidoreductase *has two distinct paths to each concept and *steroid *has one distinct path to each concept. Therefore, according to DiShIn there is only one disjunctive common ancestor, the MICA, and thus we have: *Sim*_*resnik*:*dishin *_= 0.6671. Therefore, unlike GraSM, DiShIn does not penalize *steroid *and *oxysterol *for sharing parallel interpretations. Note that we cannot apply *simGIC *to this example, since *simGIC *calculates similarity between proteins (entities), not between the concepts themselves.

### Execution time

One of the disadvantages of using GraSM was its non-linear computational complexity. GraSM improves correlation but its execution times are about 3 times higher than using Resnik, a strong limitation due to the large size of biomedical ontologies and the vast amount of entities annotated with them.

**Figure 4:**
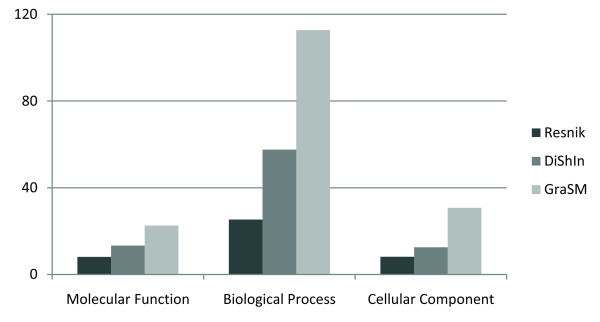
**Execution times**. Execution times for calculating the similarities of the 13,430 proteins pairs. In the y-axis we have time in minutes and in the x-axis the GO similarity measures in each subontology of GO.

Figure 4 presents the execution times, on a Quad-Core CPU at 2 GHz, of the calculation of the similarity values of all the 13,430 proteins pairs using *Sim_resnik_*, *Sim*_*resnik*:*grasm *_and *Sim*_*resnik*:*dishin*_. The Figure allows a clear performance comparison of these measures. The performance of *Sim*_*resnik*:*dishin *_is significantly closer to the performance of *Sim_resnik _*than to the performance of *Sim*_*resnik*:*grasm*_, demonstrating the superior effectiveness of DiShIn over GraSM. This was expected, given that DiShIn has an algorithmic complexity of O(n), whereas GraSM has a non-linear complexity. Thus, DiShIn improves the feasibility of the exploitation of multiple inheritance on intensive similarity calculations.

### Limitations

DiShIn is not a new semantic similarity measure. In fact, it can be considered as an add-on that efficiently incorporates multiple inheritance in the calculation of the information content that two concepts share in an ontology represented as a DAG.

DiShIn was not specifically designed to measure protein similarity either. In fact, it was adapted to do so, since protein semantic and structural similarity correlation has been a generally accepted way to assess semantic similarity approaches in the biomedical field. However, semantic and structural correlation may not be the best way to assess semantic similarity, and better gold standards, not biased by structural features, are much required, especially in the case of DiShIn, where multiple inheritance is often associated with complex entities.

DiShIn does not take advantage of the higher expressivity of more advanced ontology features than the straightforward subsumption relationships present in DAGs [31]. For semantic similarity, subsumption relationships may be enough, but as we evolve to semantic relatedness, other relationships have to be considered and additional levels of distinction between asserted and inferred hierarchies may be required.

## Conclusions

This paper presents DiShIn, a novel method for effectively exploiting multiple inheritance when calculating the shared information content between two ontology concepts. DiShIn can be easily integrated in any semantic similarity measure dependent on the information content shared by two concepts.

DiShIn was applied to GO similarity measures, and its performance was evaluated against state-of-the-art measures using an existing platform for evaluation of protein similarity measures. In this setting, DiShIn was able to improve the correlation coefficients between semantic and sequence similarity, and also reduce the computational time of the common disjunctive ancestors identification, as previously proposed by GraSM. These results represent an important contribution towards effective management of multiple inheritance in large-scale comparative studies.

As ontologies grow and interoperability between ontologies is required [32], multiple inheritance will become a prominent issue for semantic similarity measures. For example, the comparison of complex biomedical entities, such as disease and epidemiological models, is a non-trivial task due to their multiple domain features and complexity. Moreover, even the single comparison of anatomical locations remains a challenge due to the lack of a common coordinate space [33]. Thus, methods like DiShIn will certainly represent a valuable contribution for the development of multi-domain similarity measures based on an effective exploitation of multiple inheritance.

## Availability of supporting data

The data sets supporting the results of this article are available in the CESSM repository, http://xldb.di.fc.ul.pt/tools/cessm/.

## Competing interests

The authors declare that they have no competing interests.

## Authors' contributions

FMC conceived and performed the study. MJS participated in the statistical analysis and helped to write the manuscript. Both authors read and approved the final manuscript.

## References

[B1] Cross V Sudkamp T Similarity and compatibility in fuzzy set theory: assessment and applications 2002 Springer

[B2] Ehrig M Haase P Hefke M Stojanovic N Similarity for ontologies: a comprehensive framework Workshop on Ontology and Enterprise Modelling: Ingredients for Interoperability 2004

[B3] Tversky A Features of similarity Psychological review 1977 84 4 327.

[B4] Nenadić G Spasić I Ananiadou S Automatic discovery of term similarities using pattern mining COLING-02 on COMPUTERM 2002: second international workshop on computational terminology 2002 14 Association for Computational Linguistics 1 7

[B5] Pedersen T Pakhomov S Patwardhan S Chute C Measures of semantic similarity and relatedness in the biomedical domain Journal of Biomedical Informatics 2007 40 3 288 299 10.1016/j.jbi.2006.06.004 16875881

[B6] McInnes B Pedersen T Pakhomov S UMLS-Interface and UMLS-Similarity: Open source software for measuring paths and semantic similarity AMIA Annual Symposium Proceedings 2009 2009 American Medical Informatics Association 431 20351894 PMC2815481

[B7] Pesquita C Faria D Falcão AO Lord P Couto FM Semantic similarity in biomedical ontologies PLoS Computational Biology 2009 5 7 e1000443 10.1371/journal.pcbi.1000443 19649320 PMC2712090

[B8] Alvarez M Qi X Yan C A shortest-path graph kernel for estimating gene product semantic similarity Journal of Biomedical Semantics 2011 2 3 10.1186/2041-1480-2-3 PMC3161911 21801410

[B9] Lord P Stevens R Brass A Goble C Investigating semantic similarity measures across the Gene Ontology: the relationship between sequence and annotation Bioinformatics 2003 19 10 1275 1283 10.1093/bioinformatics/btg153 12835272

[B10] Washington N Haendel M Mungall C Ashburner M Westerfield M Lewis S Linking human diseases to animal models using ontology-based phenotype annotation PLoS Biol 2009 7 11 e1000247 10.1371/journal.pbio.1000247 19956802 PMC2774506

[B11] Ferreira J Couto F Semantic Similarity for Automatic Classification of Chemical Compounds PLoS computational biology 2010 6 9 e1000937 10.1371/journal.pcbi.1000937 20885779 PMC2944781

[B12] Kohler S Schulz M Krawitz P Bauer S Dolken S Ott C Mundlos C Horn D Mundlos S Robinson P Clinical diagnostics in human genetics with semantic similarity searches in ontologies The American Journal of Human Genetics 2009 85 4 457 464 10.1016/j.ajhg.2009.09.003 PMC2756558 19800049

[B13] Resnik P Using Information Content to Evaluate Semantic Similarity in a Taxonomy Proc of the 14th International Joint Conference on Artificial Intelligence 1995 448 453

[B14] Rada R Mili H Bicknell E Blettner M Development and application of a metric on semantic nets IEEE Transactions on Systems, Man and Cybernetics 1989 19 17 30 10.1109/21.24528

[B15] Couto F Silva M Coutinho P Measuring Semantic Similarity between Gene Ontology Terms Data & Knowledge Engineering 2007 61 137 152 10.1016/j.datak.2006.05.00321887854

[B16] Pesquita C Pessoa D Faria D Couto F CESSM: Collaborative Evaluation of Semantic Similarity Measures JB2009: Challenges in Bioinformatics 2009

[B17] Jiang J Conrath D Semantic Similarity Based on Corpus Statistics and Lexical Taxonomy Proc. of the 10th International Conference on Research on Computational Linguistics 1997

[B18] Lin D An information-theoretic definition of similarity Proc of the 15th International Conference on Machine Learning 1998

[B19] Dijkstra E A note on two problems in connexion with graphs Numerische mathematik 1959 1 269 271 10.1007/BF01386390

[B20] GO-Consortium The Gene Ontology (GO) database and informatics resource Nucleic Acids Research 2004 32 Database D258 D261 10.1093/nar/gkh036 PMC308770 14681407

[B21] Finn R Mistry J Tate J Coggill P Heger A Pollington J Gavin O Gunasekaran P Ceric G Forslund K The Pfam protein families database Nucleic acids research 2010 38 suppl 1 D211 19920124 10.1093/nar/gkp985 PMC2808889

[B22] Lee D Redfern O Orengo C Predicting protein function from sequence and structure Nature Reviews Molecular Cell Biology 2007 8 12 995 1005 10.1038/nrm2281 18037900

[B23] Camon E Magrane M Barrell D Lee V Dimmer E Maslen J Binns D Harte N Lopez R Apweiler R The Gene Ontology Annotation (GOA) Database: sharing knowledge in Uniprot with Gene Ontology Nucleic Acids Research 2004 32 D262 10.1093/nar/gkh021 14681408 PMC308756

[B24] Couto F Silva M Coutinho P Semantic Similarity over the Gene Ontology: Family Correlation and Selecting Disjunctive Ancestors Proc. of the ACM Conference in Information and Knowledge Management as a short paper 2005

[B25] Schlicker A Domingues FS Rahnenführer J Lengauer T A new measure for functional similarity of gene products based on Gene Ontology BMC Bioinformatics 2006 7 302 10.1186/1471-2105-7-302 PMC1559652 16776819

[B26] Azuaje F Wang H Bodenreider O Ontology-driven similarity approaches to supporting gene functional assessment Proceedings of the ISMB 2005 SIG meeting on Bio-ontologies 2005

[B27] Altschul S Madden T Schaffer A Zhang J Zhang Z Miller W Lipman D Gapped BLAST and PSI-BLAST: a new generation of protein database search programs Nucleic acids research 1997 25 17 3389 10.1093/nar/25.17.3389 9254694 PMC146917

[B28] Rosner B Fundamentals of biostatistics 2006 Duxbury Resource Center

[B29] Khatri P Drăghici S Ontological analysis of gene expression data: current tools, limitations, and open problems Bioinformatics 2005 21 18 3587 10.1093/bioinformatics/bti565 15994189 PMC2435250

[B30] Ovaska K Laakso M Hautaniemi S Fast Gene Ontology based clustering for microarray experiments BioData mining 2008 1 11 10.1186/1756-0381-1-11 19025591 PMC2613876

[B31] Aranguren M Bechhofer S Lord P Sattler U Stevens R Understanding and using the meaning of statements in a bio-ontology: recasting the Gene Ontology in OWL BMC bioinformatics 2007 8 57 10.1186/1471-2105-8-57 17311682 PMC1819394

[B32] Smith B Ashburner M Rosse C Bard J Bug W Ceusters W Goldberg L Eilbeck K Ireland A Mungall C The OBO Foundry: coordinated evolution of ontologies to support biomedical data integration Nature biotechnology 2007 25 11 1251 1255 10.1038/nbt1346 17989687 PMC2814061

[B33] Splendiani A Burger A Paschke A Romano P Marshall M Biomedical semantics in the Semantic Web Journal of Biomedical Semantics 2011 2 Suppl 1 S1 10.1186/2041-1480-2-S1-S1 21388570 PMC3105493

